# Structural insights into the mechanism of malaria-protective antibodies from the IGHV3-49/IGKV2D-29 lineage

**DOI:** 10.1063/4.0001154

**Published:** 2025-10-27

**Authors:** Monika Jain, Ian A. Wilson

**Affiliations:** 1 The Scripps Research Institute, La Jolla, California, USA; 2 The Skaggs Institute for Chemical Biology, La Jolla, California, USA

## Abstract

Malaria remains a global health concern and continues to be endemic in many tropical regions, particularly in Southeast Asia and sub-Saharan Africa. Effective vaccines and monoclonal antibody (mAb) therapies are urgently needed, especially for people living in endemic areas. Structural studies using X-ray crystallography and cryo-electron microscopy have revealed the molecular interactions of antigen-binding fragments (Fabs) from protective mAbs targeting *Plasmodium falciparum* circumsporozoite protein (PfCSP) of the liver stage sporozoites. Notably, several mAbs including 850, 1210, 399, and 311 form inter-Fab homotypic contacts upon binding the central NANP repeat region of PfCSP and demonstrate potent neutralizing activity. Among these, mAb 399, derived from the IGHV3-49/IGKV2D-29 germline, engages in head-to-head inter-Fab contacts upon CSP binding. In this study, we determined high-resolution X-ray crystal structures of additional Fabs, 7160 and 7118, both originating from the same IGHV3-49/IGKV2D-29 germline lineage, in complex with PfCSP-derived peptides. Both antibodies showed strong inhibitory activity, reducing liver-stage sporozoite burden by 90–95% in a murine challenge model, and exhibited high-affinity binding to the CSP repeat region. Strikingly, Fab 7160 formed inter-Fab homotypic interactions upon binding to an extended repeat peptide (NANP_6_), similar to the binding mode observed with Fab 399. In contrast, Fab 7118 did not engage in such contacts. Structural analysis suggests that variations in the complementarity-determining region (CDRH3), particularly in Fab 7118 which features a longer loop may introduce steric hindrance and electrostatic repulsion, thereby preventing homotypic Fab-Fab interactions that have been found to be beneficial in mouse models of protection for other antibodies.

